# Swedish snuff (snus) and risk of cardiovascular disease and mortality: prospective cohort study of middle-aged and older individuals

**DOI:** 10.1186/s12916-021-01979-6

**Published:** 2021-05-07

**Authors:** Olga E. Titova, John A. Baron, Karl Michaëlsson, Susanna C. Larsson

**Affiliations:** 1 Department of Surgical Sciences, Unit of Medical Epidemiology, The EpiHub, Uppsala University, Dag Hammarskjölds väg 14 B, 75185 Uppsala, Sweden; 2 Department of Medicine, University of North Carolina School of Medicine, Chapel Hill, NC USA; 3 Department of Epidemiology, Gillings School of Global Public Health, University of North Carolina, Chapel Hill, NC USA; 4 Unit of Cardiovascular and Nutritional Epidemiology, Institute of Environmental Medicine, Karolinska Institutet, Stockholm, Sweden

**Keywords:** Cardiovascular disease, Snus, Snuff, Smokeless tobacco, Mortality

## Abstract

**Background:**

Cigarette smoking is a well-known risk factor for cardiovascular disease (CVD), but whether smokeless tobacco such as snuff is associated with the risk of CVD is still unclear. We investigated the association of the use of Swedish oral moist snuff (snus) with a broad range of CVDs and CVD mortality.

**Methods:**

We used data from a population-based cohort of 41,162 Swedish adults with a mean baseline age of 70 (56–94) years who completed questionnaires regarding snus use and other lifestyle habits and health characteristics. Participants were followed up for incident cardiovascular outcomes and death over 8 years through linkage to the Swedish National Patient and Death Registers. Hazard ratios (HR) were estimated by Cox proportional hazards regression. We conducted analyses among all subjects as well as among never smokers to reduce residual confounding from smoking.

**Results:**

After adjustment for smoking and other confounders, snus use was not associated with myocardial infarction, heart failure, atrial fibrillation, aortic valve stenosis, abdominal aortic aneurysm, stroke, or CVD mortality. However, in never smokers, snus use was associated with a statistically significant increased risk of total and ischemic stroke (HRs [95% confidence intervals] = 1.52 [1.01–2.30] and 1.63 [1.05–2.54], respectively) and non-significantly positively associated with some other CVDs.

**Conclusions:**

In this middle-aged and elderly Swedish population, current Swedish snus use was not associated with the risk of major heart and valvular diseases, abdominal aortic aneurysm, or CVD mortality in the entire study population, but was linked to an increased risk of stroke in never smokers.

**Supplementary Information:**

The online version contains supplementary material available at 10.1186/s12916-021-01979-6.

## Background

Cigarette smoking is one of the leading causes of morbidity and premature death worldwide [1]. The adverse effect of cigarette smoking on cardiovascular health has been well documented in prospective cohort studies [1–7]. A recent Mendelian randomization study provided further evidence for causal association between smoking and a broad range of cardiovascular diseases (CVDs), including coronary artery disease, heart failure (HF), abdominal aortic aneurysm (AAA), and ischemic stroke [8]. Consequently, strategies to reduce cigarette smoking are of great importance globally. There is considerable discussion regarding the effectiveness of nicotine replacement therapy, and safety of the alternative nicotine delivery products such as smokeless tobacco, especially among those who are less likely or unwilling to quit smoking [9, 10].

Smokeless tobacco products may vary considerably in composition and the amount of potentially toxic compounds [11]. Oral moist snuff (snus) is commonly used in Sweden and some other Scandinavian countries as well as in the USA. Swedish snus is used loose or in a portion-packed form (sachets) and is typically placed between the lip and the gum [11]. Compared to the late 1980s, there was an increase in snus consumption in Sweden, accompanied by a considerable decline in prevalence of cigarette smoking [12]. During 2016–2017, 22% of Swedish men and 5% of women were daily snus users and 11% of the population were daily smokers according to the central statistical agency in Sweden [12]. Despite the absence of harmful combustion products, the amount of nicotine in smokeless tobacco is comparable to that in cigarettes and the peak blood concentration of nicotine in users is similar to those observed in cigarette smokers [13, 14]. Nicotine raises heart rate and blood pressure regardless of the route of administration [15–17]. However, the long-term effect of moist snuff on the cardiovascular system has not been thoroughly studied and results remain inconsistent.

Previous research regarding the association between snuff use and CVD has mainly focused on CVD-specific mortality, incidence of ischemic heart disease, myocardial infarction (MI), and stroke [18–20]. An increased risk of CVD or CVD-related death was demonstrated in some [21, 22], but not all, prospective studies [23–25]. Studies of the association between snuff use and other CVDs, such as heart failure (HF) [21], atrial fibrillation (AF) [25], aortic valve stenosis (AVS), and AAA are scarce or absent.

The aim of this study was to investigate the associations of Swedish snus use with specific CVD events, including MI, HF, AF, AVS, AAA, and stroke, as well as CVD mortality in a cohort of 41,162 Swedish men and women (56–94 years of age). Cigarette smoking has previously been shown to be significantly associated with each of these CVDs [2–7] except AF [26] in this study population, but the association between snus use and CVD was not investigated.

## Methods

### Study population

The data from the National Research Infrastructure SIMPLER (Swedish Infrastructure for Medical Population-based Life-course Environmental Research) was used in this analysis. A detailed description can be found elsewhere (https://www.simpler4health.se). Information on lifestyle and other risk factors for CVD and other diseases was obtained with structured questionnaires in 2008/2009. In the present analysis, we excluded individuals who died or had a study endpoint prior to July 1, 2009, and those who had missing information on snus use or cigarette smoking (Additional file 1). This left 41,162 eligible participants (18,027 women and 23,135 men) with a mean baseline age of 70 (56–94) years. In the main analysis of each CVD outcome, we excluded participants with a diagnosis of the corresponding specific CVD before start of a follow-up (e.g., those with MI before baseline were excluded from the analysis of MI; those who were diagnosed with any stroke before baseline, were excluded from the analyses of stroke and stroke types), as ascertained through linkage to the Swedish National Patient Register from 1987 and based on the International Classification of Diseases (ICD)-9 and ICD-10 codes. In the analysis of overall CVD mortality, those who were diagnosed with any CVD before the start of a follow-up were excluded. The number of prevalent CVD cases excluded in each analysis is shown in Additional file 1**.**

### Assessment of snus use, potential confounders, and intermediates

In 2008/2009, participants completed a Health questionnaire and Diet and lifestyle questionnaire that included information about snus use, smoking status, alcohol consumption, educational attainment, weight, height, physical activity, and history of diabetes, hypertension, and hypercholesterolemia. Participants were asked if they used snus regularly (more than 5 portions of snus/week) with the following options: no; yes, currently; yes, in the past. In addition, former snus users (*n* = 2946, 7% of the entire cohort) indicated how many years ago they stopped using snus. As most ever using participants stopped using snus long before the start of the follow-up (median; quartile 1–quartile 3; 20 (7–28) years), former snus users and non-users were combined into one group for analysis. Participants were also asked if they smoked cigarettes regularly (more than 5 cigarettes/week) with the following options: no; yes, currently; yes, in the past. Participants who answered that they never smoked cigarettes regularly were defined as never smokers. A participant was considered to have a history of diabetes if he/she reported having diabetes and/or diabetes treatment.

### Case ascertainment and follow-up

Incident cases of CVDs that occurred after the start of follow-up were ascertained through linkage with the Swedish National Patient Register (covering both in- and out-patients) and the Cause of Death Register using the unique personal identity number assigned to each Swedish resident and classified according to the ICD 10th Revision codes. The endpoints in the present study were acute MI (I21), HF (I50 and I11.0), AF (I48), AVS (I35.0 and I35.2), AAA (I71.3 and I71.4), ischemic stroke (I63), intracerebral hemorrhage (I61), subarachnoid hemorrhage (I60), unspecified stroke (I62), and CVD mortality (I00–I99) as the primary cause of death. Participants were followed up from July 1, 2009, to the first date of diagnosis of specific CVD or CVD-mortality, death from any cause, or December 31, 2017, whichever occurred first.

### Statistical analysis

Descriptive data are presented as mean (standard deviation) for continuous variables and as the number of participants (%) for categorical variables. Numerical data were analyzed with the Mann-Whitney *U* test. The Pearson chi-square test was used to analyze group differences for categorical variables. Cox proportional hazards regression models were used to obtain hazard ratios (HR) with 95% confidence intervals (CI) with age as the time scale and adjusted for sex (as a stratification variable) in the basic model. Snus use status was classified as non-use (never used snus regularly or former users) and current snus use at baseline.

First, we investigated the associations of snus use and CVD risk in the population-based cohort, including all participants in the analysis. In a first multivariable model, we further adjusted for education (less than high school, high school, or university), smoking status (never, former, current smokers), walking/bicycling (never/seldom; < 20 min/day; 20–40 min/day; > 40 min/day), alcohol intake (never drinkers; past or current drinkers of < 1 drink/week; 1–< 7 drinks/week; 7–< 15 drinks/week; 15–21 drinks/week; > 21 drinks/week), and exercise (almost never; < 1 h/week; 1 h/week; 2–3 h/week; 4–5 h/week; ≥ 5 h/week). In a second multivariable model, we further adjusted for potential intermediates of the association of snus use with CVD risk, including body mass index (weight divided by the square of height; < 22.5, 22.5–24.9, 25.0–29.9, or ≥ 30 kg/m^2^) and history of diabetes (no/yes), hypertension (no/yes), and hypercholesterolemia (no/yes). For categorical variables, the category with the lowest value was treated as the reference group, except for body mass index where normal weight (22.5–24.9 kg/m^2^) was used as the reference.

The proportion of missing data on the potential confounders/intermediates used in the main analysis was less than 5%. A separate category (“missing”) was created for each variable containing missing values. To reduce confounding from cigarette smoking, we conducted a separate analysis of the association between snus use and CVD risk among individuals who reported never smoking regularly. Proportional hazard assumptions were assessed by Schoenfeld’s test. Potential confounders were selected using directed acyclic graphs (DAGs) [27] based on our a priori knowledge of the relationships among potential confounders, intermediate variables, exposure, and outcome variables, as well as on existing information regarding factors associated with CVD and tobacco consumption [28, 29]. With regard to HF, we performed a sensitivity analysis additionally excluding individuals with a diagnosis of MI before the start of a follow-up. In addition, we conducted analyses using never snus users as the reference group; as well as analyses including only men. All statistical tests were two sided. All statistical analyses were performed using Stata version 15.1 (StataCorp, College Station, TX, USA).

## Results

Baseline characteristics of study participants according to snus use are shown in Table 1. Compared with non-users, current snus users were younger, had lower educational attainment, were more likely to be men, had higher alcohol intake, were more likely to be former or current cigarette smokers, were less physically active, had higher BMI, and were less likely to report hypertension at the baseline (*p* < 0.05). As the majority of snus users were men, we compared these baseline characteristics for men non-users and men snus users. The results were similar as for the entire cohort, except for history of hypertension which did not differ between non-users and snus users (data not shown).

**Table 1:** Baseline characteristics according to snus use in 41,162 Swedish middle-aged and elderly participants, 2009–2017

Characteristics*	Snus use	
	Nonusers	Users
**Number of participants,** *n* (% of total)	38,862 (94.4)	2300 (5.6)
**Age at baseline, years**, mean (SD)	69.8 (8.0)	65.5 (6.7)
**Men**, *n* (%)	20,988 (54.0)	2147 (93.4)
**Education**, *n* (%)		
≤ 9 years	11,733 (30.2)	716 (31.1)
10–12 years	18,104 (46.6)	1164 (50.6)
> 12 years	89,291 (23.0)	412 (17.9)
Unknown	96 (0.25)	8 (0.35)
**Cigarette smoking status,** *n* (%)		
Non-smokers	21,099 (54.3)	418 (18.2)
Former smokers	14,411 (37.1)	1590 (69.1)
Current smokers	3352 (8.6)	292 (12.7)
**Alcohol intake**, *n* (%)		
Never drinkers	3689 (9.5)	18 (0.8)
Past or current drinkers of < 1 drink/week	8198 (21.1)	328 (14.3)
1–< 7 drinks/week	18,605 (47.9)	1148 (49.9)
7–< 15 drinks/week	7085 (18.2)	644 (28.0)
15–21 drinks/week	957 (2.5)	116 (5.0)
> 21 drinks/week	328 (0.8)	46 (2.0)
**Walking/bicycling**, *n* (%)		
Never/seldom	2401 (6.2)	173 (7.5)
< 20 min/day	6652 (17.1)	514 (22.4)
20–40 min/day	16,215 (41.7)	869 (37.8)
> 40 min/day	13,031 (33.5)	719 (31.3)
Unknown	563 (1.5)	25 (1.1)
**Exercise,** *n* (%)		
Almost never	20,868 (53.7)	1515 (65.9)
< 1 h/week	4694 (12.1)	256 (11.1)
1 h	6623 (17.0)	234 (10.2)
2–3 h	5020 (12.9)	211 (9.2)
4–5 h	705 (1.8)	44 (1.9)
≥ 5 h/week	268 (0.7)	8 (0.4)
Unknown	684 (1.8)	32 (1.4)
**Body mass index category**, kg/m^2^, *n* (%)		
22.5–24.9	10,035 (25.8)	523 (22.7)
< 22.5	6553 (16.9)	227 (9.9)
25.0–29.9	16,053 (41.3)	1109 (48.2)
≥ 30.0	4990 (12.8)	375 (16.3)
Unknown	1231 (3.2)	66 (2.9)
**Hypertension**, *n* (%)	16,214 (41.7)	887 (38.6)
**Hypercholesterolemia**, *n* (%)	9832 (25.3)	589 (25.6)
**Diabetes**, *n* (%)		
No	34,188 (88.0)	2005 (87.2)
Yes	3560 (9.2)	225 (9.8)
Unknown	1114 (2.9)	70 (3.0)

The number of incident CVDs and CVD-related deaths during up to 8 years of follow-up is shown in Table 2. In age- and sex-adjusted analysis, current snus use was associated with increased risk of AF and AAA compared with non-use, but these associations did not remain after adjustment for cigarette smoking (the major confounder) and other risk factors (Table 2). There was no association between snus use and the other CVDs.

**Table 2:** Hazard ratios (95% confidence intervals) of CVDs according to snus use in the entire study population of Swedish adults, 2009–2017

	Snus use	
Outcome and model (total number)	Nonusers	Users
**Myocardial infarction (*N*** **= 38,844)**		
Total number of cases	1610	97
Age and sex-adjusted model	1.00 (reference)	1.01 (0.82–1.25)
Multivariable model 1^†^	1.00 (reference)	0.95 (0.77–1.18)
Multivariable model 2^††^	1.00 (reference)	0.96 (0.78–1.19)
**Heart failure (*N*** **= 40,326)**		
Total number of cases	1990	96
Age and sex-adjusted model	1.00 (reference)	1.15 (0.94–1.42)
Multivariable model 1^†^	1.00 (reference)	0.99 (0.80–1.23)
Multivariable model 2 ^††^	1.00 (reference)	1.00 (0.81–1.23)
**Atrial fibrillation** (***N*** **= 38,044**)		
Total number of cases	3991	234
Age and sex-adjusted model	1.00 (reference)	**1.18 (1.03–1.35)**
Multivariable model 1^†^	1.00 (reference)	1.11 (0.97–1.27)
Multivariable model 2^††^	1.00 (reference)	1.11 (0.97–1.28)
**Aortic valve stenosis (*N*** **= 40,873)**		
Total number of cases	421	19
Age and sex-adjusted model	1.00 (reference)	0.86 (0.54–1.37)
Multivariable model 1^†^	1.00 (reference)	0.82 (0.51–1.32)
Multivariable model 2^††^	1.00 (reference)	0.83 (0.52–1.33)
**Abdominal aortic aneurysm (*N*** **= 40,853)**		
Total number of cases	480	55
Age and sex-adjusted model	1.00 (reference)	**1.37 (1.03–1.81)**
Multivariable model 1^†^	1.00 (reference)	1.06 (0.79–1.40)
Multivariable model 2^††^	1.00 (reference)	1.07 (0.80–1.42)
Total stroke* (***N*** **= 39,399**)		
Total number of cases	2071	105
Age and sex-adjusted model	1.00 (reference)	1.09 (0.89–1.34)
Multivariable model 1^†^	1.00 (reference)	1.04 (0.85–1.27)
Multivariable model 2 ^††^	1.00 (reference)	1.04 (0.85–1.27)
**Total ischemic stroke (*N*** **= 39,399)**		
Total number of cases	1726	88
Age and sex-adjusted model	1.00 (reference)	1.12 (0.90–1.39)
Multivariable model 1^†^	1.00 (reference)	1.05 (0.84–1.31)
Multivariable model 2^††^	1.00 (reference)	1.05 (0.84–1.32)
**Total hemorrhagic stroke (*N*** **= 39,399)**		
Total number of cases	317	20
Age and sex-adjusted model	1.00 (reference)	1.21 (0.76–1.93)
Multivariable model 1^†^	1.00 (reference)	1.16 (0.73–1.86)
Multivariable model 2 ^††^	1.00 (reference)	1.15 (0.72–1.85)
**CVD mortality (*N*** **= 34,355)**		
Total number of deaths	1333	58
Age and sex-adjusted model	1.00 (reference)	1.17 (0.89–1.53)
Multivariable model 1^†^	1.00 (reference)	1.03 (0.78–1.35)
Multivariable model 2^††^	1.00 (reference)	1.02 (0.78–1.35)

*CI* confidence interval, *HR* hazard ratio. Participants with a diagnosis of the corresponding CVD before the start of a follow-up were excluded from the disease-specific analysis. In the analysis of overall CVD mortality, those who were diagnosed with CVD before the start of a follow-up were also excluded

*Includes ischemic stroke, intracerebral hemorrhage, subarachnoid hemorrhage, and undefined type of stroke

^†^The Cox proportional hazards regression model was adjusted for age (underlying time scale), sex (as a stratification variable), education, cigarette smoking, alcohol consumption, walking/bicycling, and exercise

^††^The Cox proportional hazards regression model was further adjusted for potential intermediates: body mass index (categorical), and history of hypertension, hypercholesterolemia, and diabetes

**Fig. 1:**
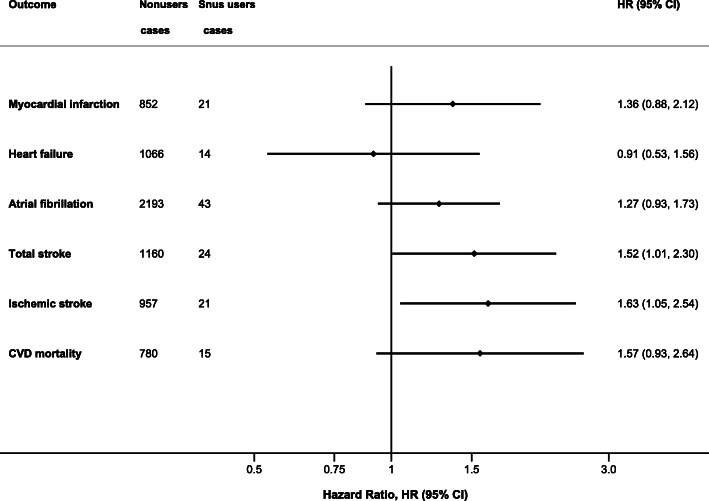
Multivariable HRs (95% CI) of cardiovascular diseases (CVD) and CVD death according to snus use in participants who reported never smoking cigarettes regularly. The Cox proportional hazards regression models were adjusted for age (underlying time scale), sex (as a stratification variable), education, alcohol consumption, walking/bicycling, exercise, body mass index, and history of hypertension, hypercholesterolemia, and diabetes. CI, confidence interval; HR, hazard ratio

After restriction of the database to never-smokers, current snus use was associated with a significant increased risk of total stroke and ischemic stroke (multivariable HRs [95% CIs] = 1.52 [1.01–2.30] and 1.63 [1.05–2.54], respectively) (Table 3, Fig. 1). In age- and sex-adjusted analysis, current snus use was associated with an increased risk of CVD death, but this association did not remain statistically significant after adjustment for potential confounders. In addition, a trend for an increased risk of MI and AF in snus users was observed, but results did not attain statistical significance (Table 3**,** Fig. 1). The results for AVS, AAA, and hemorrhagic stroke were not shown due to the small number of cases in the group of snus users (*n* < 5).

**Table 3:** Hazard ratios (95% confidence intervals) of CVDs according to snus use in Swedish adults who reported never smoking cigarettes regularly, 2009–2017

	Snus use	
Outcome and model (total number)	Nonusers	Users
**Myocardial infarction (*N*** **= 20,501)**		
Total number of cases	852	21
Age and sex-adjusted model	1.00 (reference)	1.35 (0.87–2.10)
Multivariable model 1^†^	1.00 (reference)	1.36 (0.87–2.11)
**Heart failure (*N*** **= 21,130)**		
Total number of cases	1066	14
Age and sex-adjusted model	1.00 (reference)	1.04 (0.61–1.78)
Multivariable model 1^†^	1.00 (reference)	0.92 (0.54–1.57)
**Atrial fibrillation** (***N*** **= 19,852**)		
Total number of cases	2193	43
Age and sex-adjusted model	1.00 (reference)	1.33 (0.98–1.81)
Multivariable model 1^†^	1.00 (reference)	1.29 (0.95–1.75)
Total stroke* (***N*** **= 20,620**)		
Total number of cases	1160	24
Age and sex-adjusted model	1.00 (reference)	**1.57 (1.04–2.37)**
Multivariable model 1^†^	1.00 (reference)	**1.53 (1.02–2.32)**
**Total ischemic stroke (*N*** **= 20,620)**		
Total number of cases	957	21
Age and sex-adjusted model	1.00 (reference)	**1.69 (1.09–2.63)**
Multivariable model 1^†^	1.00 (reference)	**1.65 (1.06–2.57)**
**CVD mortality** (***N*** **= 18,189**)		
Total number of deaths	780	15
Age and sex-adjusted model	1.00 (reference)	**1.70 (1.01–2.87)**
Multivariable model 1^†^	1.00 (reference)	1.58 (0.94–2.67)

*CI* confidence interval, *HR* hazard ratio. Participants with a diagnosis of the corresponding CVD before the start of a follow-up, were excluded from the disease-specific analysis. In the analysis of overall CVD mortality, those who were diagnosed with CVD before the start of a follow-up were also excluded

*Includes ischemic stroke, intracerebral hemorrhage, subarachnoid hemorrhage, and undefined type of stroke

^†^The Cox proportional hazards regression model was adjusted for age (underlying time scale), sex (as a stratification variable), education, alcohol consumption, walking/bicycling, and exercise

In a sensitivity analysis, the results for HF were similar after additional exclusion of individuals with MI before baseline (HR 1.05, 95% CI 0.84–1.32, multivariable model 2 in the entire sample; HR 0.81, 95% CI 0.44–1.49, multivariable model 2 in never smokers). The results for current snus users were similar for all CVD outcomes when using “never snus users” as a reference group (data not shown). In addition, we performed analyses including only men and found similar results to those presented in Tables 2 and 3 (data not shown).

## Discussion

In this study, we investigated the association between snus use and the subsequent risk of several CVDs and CVD mortality in a cohort of middle-aged and older men and women. Findings from the analyses adjusted for smoking that are based on the entire cohort, do not support a detrimental effect of snus use on the risk of major heart and valvular diseases, AAA, or CVD mortality. However, in never smokers, snus use was associated with an increased risk of stroke, particularly ischemic stroke, and non-significantly associated with increased risk of myocardial infarction, atrial fibrillation, and CVD death.

### Comparisons with other studies

Several health hazards associated with smokeless tobacco use have previously been documented, including oral cancer [30], CVD [31], and all-cause and cause-specific mortality [32]. Our findings of no significant association between snus use and incidence of MI and AF are in agreement with previous reports [18, 25, 33, 34]. For example, in a pooled analysis of eight Swedish prospective cohort studies, current snus use was not related to risk of acute MI [18]. However, long-term use of snus was associated with an increased relative risk of fatal MI, especially among heavy users, in a cohort of nonsmoking male construction workers (mean baseline age of 31.5 years and 19 years of follow-up) [33]. Similar to our results, snus use was not associated with AF risk in a pooled analysis of 7 Swedish prospective cohorts of never-smoking men [25].

In contrast to our finding, a previous Swedish study demonstrated that snus use was associated with a higher risk of HF in elderly men after adjusting for smoking status (*n* = 1076; HR, 2.08; 95% CI, 1.03–4.22) and in younger never-smoking male construction workers (*n* = 118,425; HR, 1.28; 95% CI, 1.00–1.64) [21]. Baseline information in this previous study was collected in 1991–1995 (cohort of elderly men) vs 2008–2009 in our study and might be related to differences in lifestyle and snus composition during these time periods. For example, a higher proportion of current cigarette smokers was observed among elderly men snus users [21] compared to our study. Moreover, the amount of some potentially toxic compounds such as tobacco-specific nitrosamines and some polycyclic aromatic hydrocarbons (PAH) was substantially reduced in Swedish snus in the early 2000s; and the GothiaTek standard that regulates the content of such undesired components and specifies manufacturing standards, was introduced in 2000 [11]. In addition, in the analysis of elderly men [21], the association of snus use with HF risk among never cigarette smokers was not examined, likely resulting in residual confounding from smoking. Another study based on two US prospective cohorts reported higher CVD mortality among men who currently used chewing tobacco or snuff compared with non-users [22]. In the present study, an increased risk of CVD death among snus users with no history of cigarette smoking was observed after adjustment for age and sex only, but the association did not persist after adjustment for other confounders.

We are not aware of any previous study on snuff use in relation to risk of AVS or AAA. In our study population, cigarette smoking was previously shown to be significantly associated with an increased risk of these outcomes [3, 7], with a particularly strong association with AAA (almost 7-fold and 11-fold increased risks in heavy smoking men and women, respectively) [7].

Our findings of a positive association between current snus use and risk of total stroke and ischemic stroke in never smokers indicate that nicotine per se may contribute to the pathophysiology of stroke. This is in line with previous reports of an increased risk of stroke fatality among snuff or chewing tobacco users compared to non-users [22, 23, 35]. However, a recent meta-analysis of studies conducted in the USA and Sweden, demonstrated an increased risk of stroke among USA but not in Swedish smokeless tobacco users [19]. Thus, several Swedish studies have found no association between the use of snus and incident stroke in non-smokers or in the entire sample analysis adjusted for smoking status [23, 24, 34, 36]. Importantly, these prospective cohort studies included younger participants compared to the present study. For example, in the pooled analysis of eight Swedish prospective cohort studies, including never-smoking men, no association between the use of snus and the risk of overall stroke or any stroke types were observed [23]. The baseline mean age in this pooled study was 35 years vs 70 years in our study. The possible explanation that we see the effect of snus use on the risk of stroke only in never smokers is that the majority of snus users in our study are former smokers (69%). Cigarette smoking is a well-established strong risk factor for CVDs and may mask the effect of snus in the analysis based on the entire cohort, i.e. residual confounding takes place. In addition, survival bias could occur especially in such cohort of middle-aged and elderly participants, i.e., some former and current smokers could die before the baseline or be excluded from the analysis due to a cardiovascular event prior baseline.

As in cigarettes, nicotine is the main alkaloid in snuff. A number of animal and human studies indicated that nicotine may contribute to CVD via multiple pathways [37–40]. For example, hypertension is the main risk factor for stroke. While epidemiological findings on snuff use and hypertension are inconclusive [13, 16], experimental evidence suggests that nicotine per se (e.g., nicotine infusion) and smokeless tobacco use may acutely increase blood pressure and heart rate as well as cause endothelial dysfunction in healthy volunteers [38–41]. These adverse effects of nicotine might be related to nicotine-induced imbalance in the homeostasis of the renin-angiotensin system, an important regulatory peptide hormone component of the cardiovascular system [42]. In addition, nicotine may play a role in the enhancement of arterial wave reflection to the aorta, an indirect measure of arterial stiffness and an important determinant of central blood pressure [43]. Nicotine may also induce cardiac arrhythmias [44], which increase the risk of ischemic stroke, specifically the cardioembolic stroke subtype. Unfortunately, we did not have information on ischemic stroke subtypes and thus could not assess whether the association between snus use and stroke risk was confined to cardioembolic stroke. In vivo studies have also demonstrated that chronic nicotine exposure may have an adverse effect on cerebral blood flow and blood-brain barrier and enhances the degree of brain damage following an ischemic insult [45, 46].

Although the amount of nicotine in smokeless tobacco (e.g., snuff, chewing tobacco) is similar to that in cigarettes, the absorption is slower in smokeless tobacco [13]. In addition, unlike cigarette smoking, snuff does not consist of harmful combustion products, and some tobacco components can be better absorbed through the airways than through the oral mucosa [47]. This may at least partially explain less adverse effects of snuff on the circulatory system compared to cigarette smoking. The effect of other snuff constituents on the cardiovascular system is unknown and is likely minor [13].

### Strength and limitations

Important strengths of our study are large sample size; a broad range of CVD outcomes, objectively assessed through linkage to nationwide population-based registers; complete case identification and no loss to follow-up; and the ability to adjust for important confounders. In the analyses, adjusting for smoking status, we cannot rule out residual confounding by smoking and this model rather evaluates the impact of snus use on a population level. To reduce residual confounding by smoking, we performed analyses of the association between snus using and CVD risk in never smokers.

Several limitations, however, apply to the present study. We cannot rule out that survival bias may have affected our results. For example, in the entire group, smokers may have died from other causes or had a cardiovascular event before baseline and therefore were not included in the present analyses. Another limitation is that the proportion of snus users was relatively small. Duration of snus use (and of smoking before baseline) was not available and some current snus users might have quit or changed the amount of snus consumed during the 8-year long follow-up period. In addition, in current smokers, we were unable to account for the number of cigarettes smoked per day. The incidence of AAA, AVS, and hemorrhagic stroke in current snus users who reported never smoking cigarettes regularly was low, and therefore, results for these outcomes were not presented. Similarly, the small number of fatal CVD cases prevented us from investigating the relationship of snus use and case fatality for specific CVDs (e.g., fatal MI or stroke). In addition, the proportion of women snus users was small. In the analyses confined to never smokers, we could not rule out a modestly higher risk of MI, AF, and CVD death in snus users. Also, our results might be not generalizable to other populations due to differences in smokeless tobacco composition and form (e.g., chewing tobacco). Finally, in view of the observational nature of this study, we cannot rule out residual and unmeasured confounding.

Further large-scale health studies of snuff use are needed. Such research concerning snuff use in relation to the development of CVDs should address sex differences, dose and recency of use, and age effects of snuff use.

## Conclusions

Results from this prospective cohort study of middle-aged and older Swedish adults indicate that snus use is not associated with the risk of major heart and valvular diseases, AAA, or CVD mortality in the entire study population, but is linked to an increased risk of stroke in never smokers. A potential harmful effect of snus use on other CVDs in never smokers cannot be ruled out and needs further study. The results of this study are mainly applicable to the Swedish type of snuff (snus).

## Supplementary Information

**Additional file 1.** Flow-chart.

## Data Availability

The data that support the findings of this prospective cohort study are available upon application to the Swedish Infrastructure for Medical Population-based Life-course Environmental Research (SIMPLER; https://www.simpler4health.se).

## Abbreviations

AAA: Abdominal aortic aneurysm
AF: Atrial fibrillation
AVS: Aortic valve stenosis
BMI: Body mass index
CI: Confidence interval
CVD: Cardiovascular diseases
HF: Heart failure
HR: Hazard ratios
MI: Myocardial infarction
