# Supplementary figures and images for: Swedish snuff (snus) and risk of cardiovascular disease and mortality: prospective cohort study of middle-aged and older individuals
Source: BMC Med. 2021 May 7;19:111. doi: 10.1186/s12916-021-01979-6 (PMC8103653; doi:10.1186/s12916-021-01979-6)

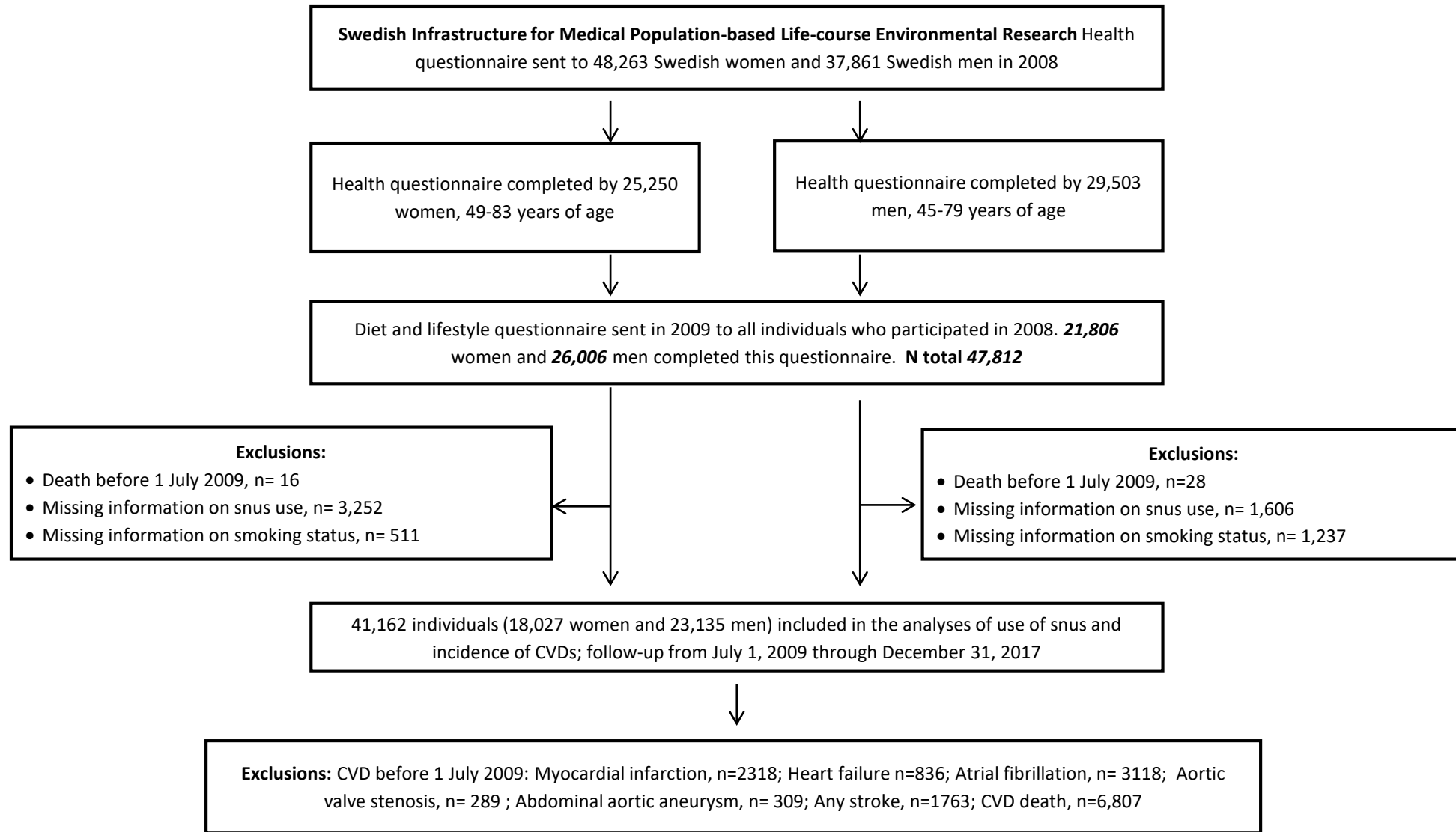

**Additional file 1.** Flow chart of study participants

Supplement: Supplementary file 1 — Additional file 1. Flow-chart. [file 12916_2021_1979_MOESM1_ESM.pdf]
